# Functional Characterization of the Osteoarthritis Genetic Risk Residing at *ALDH1A2* Identifies rs12915901 as a Key Target Variant

**DOI:** 10.1002/art.40545

**Published:** 2018-08-23

**Authors:** Colin Shepherd, Dongxing Zhu, Andrew J. Skelton, Jennifer Combe, Harrison Threadgold, Linyi Zhu, Tonia L. Vincent, Paul Stuart, Louise N. Reynard, John Loughlin

**Affiliations:** ^1^ Newcastle University Newcastle upon Tyne UK; ^2^ Newcastle University, Newcastle upon Tyne, UK and Guangzhou Institute of Cardiovascular Disease, The Second Affiliated Hospital, and Guangzhou Medical University Guangzhou China; ^3^ Arthritis Research UK Centre for OA Pathogenesis University of Oxford Oxford UK; ^4^ Newcastle University Teaching Hospitals NHS Trust Freeman Hospital Newcastle upon Tyne UK

## Abstract

**Objective:**

To identify the functional single‐nucleotide polymorphisms (SNPs) and mechanisms conferring increased risk of hand osteoarthritis (OA) at the *ALDH1A2* locus, which is a retinoic acid regulatory gene.

**Methods:**

Tissue samples from 247 patients with knee, hip, or hand OA who had undergone joint surgery were included. RNA‐sequencing analysis was used to investigate differential expression of *ALDH1A2* and other retinoic acid signaling pathway genes in cartilage. Expression of *ALDH1A2* in joint tissues obtained from multiple sites was quantified using quantitative reverse transcription–polymerase chain reaction. Allelic expression imbalance (AEI) was measured by pyrosequencing. The consequences of *ALDH1A2* depletion by RNA interference were assessed in primary human chondrocytes. In silico and in vitro analyses were used to pinpoint which, among 62 highly correlated SNPs, could account for the association at the locus.

**Results:**

*ALDH1A2* expression was observed across multiple joint tissue samples, including osteochondral tissue from the hand. The expression of *ALDH1A2* and of several retinoic acid signaling genes was different in diseased cartilage compared to non‐diseased cartilage, with *ALDH1A2* showing lower levels in OA cartilage. Experimental depletion of *ALDH1A2* resulted in changes in the expression levels of a number of chondrogenic markers, including *SOX9*. In addition, reduced expression of the OA risk–conferring allele was witnessed in a number of joint tissues, with the strongest effect in cartilage. The intronic SNP rs12915901 recapitulated the AEI observed in patient tissues, while the Ets transcription factors were identified as potential mediators of this effect.

**Conclusion:**

The *ALDH1A2* locus seems to increase the risk of hand OA through decreased expression of *ALDH1A2* in joint tissues, with the effect dependent on rs12915901. These findings indicate a mechanism that may now be targeted to modulate OA risk.

Osteoarthritis (OA) is a common age‐related disease that is characterized by the focal loss of cartilage, and that is accompanied by pathologic alterations to additional joint tissues 1. There are no disease‐modifying drugs for OA, with arthroplasty of the hips and knees being a common procedure.

Epidemiologic studies have reported an OA heritability of >40% at individual skeletal sites 2, while data sets from candidate‐gene and genome‐wide association studies have revealed that OA is polygenic, with the evidence indicating that there is no risk‐conferring loci of large singular impact. As such, the disease is genetically complex and multifactorial.

To date, the OA loci identified are typically located within regions of the genome harboring genes encoding proteins involved in joint development, maturation, or homeostasis and that tend to contribute to disease susceptibility only at particular skeletal sites 2, 3, 4, 5. An example of this is the hand OA–associated single‐nucleotide polymorphisms (SNPs) in *ALDH1A2* 6. *ALDH1A2* codes for the enzyme RALDH2, which synthesizes the morphogen retinoic acid. Studies have shown that retinoic acid has pivotal roles in the development and maintenance of the skeleton, with its effect mediated at the gene transcriptional level 7, 8.

The association of *ALDH1A2* with OA risk was discovered in Icelanders and replicated in cohorts from the UK and Netherlands in studies using proxy SNPs rs3204689 and rs4238326. The greatest odds ratio in the combined analysis was 1.46 for rs3204689 (*P* = 1.1 × 10^−11^). None of the SNPs that correlate (r^2^ > 0.8) with rs3204689 or rs4238326 are nonsynonymous, implying that the association acts by modulating gene expression as a quantitative trait locus (eQTL). The presence of an eQTL operating on *ALDH1A2* was confirmed using the 3′‐untranslated region (3′‐UTR) SNP rs3204689. An average 17.4% excess expression of *ALDH1A2* messenger RNA (mRNA) from the non‐risk G allele, relative to the risk‐conferring C allele, was reported in knee and hip cartilage samples 6. This is equivalent to a 15% reduction in *ALDH1A2* expression in the presence of the risk allele.

In this study, we aimed to expand the allelic expression imbalance (AEI) analysis into other synovial joint tissues and to the trapezium of patients who had undergone a trapeziectomy due to hand OA. We carried out a broad analysis of the expression of *ALDH1A2* and genes involved in the retinoic acid pathway. Finally, we performed experiments to identify a SNP or SNPs in the association signal that recapitulate the AEI effect observed in patient tissues and which could, therefore, be functional candidate SNPs.

## Patients and methods

**Patients.** Joint tissue samples were obtained through 2 centers in the UK, Newcastle and Oxford. The Newcastle collection was undertaken essentially as previously described 9. The Newcastle and North Tyneside Research Ethics Committee granted ethics approval for the collection. Each donor provided verbal and written informed consent (REC reference no. 14/NE/1212). Samples were collected from 1) patients with primary hip or knee OA who had undergone joint replacement surgery, 2) patients with primary hand OA who had undergone trapeziectomy, and 3) patients who had undergone hip replacement due to a neck‐of‐femur (NOF) fracture. For patients with hand OA, cartilage could not be readily separated from fractured subchondral bone; therefore these samples comprised subchondral bone with attached cartilage (i.e., osteochondral samples). Tissue preparation and grinding was performed as described previously 10.

DNA and RNA were extracted from the cartilage, bone, and trapezieum samples using TRIzol reagent (Life Technologies). For the synovium and fat pad, DNA and RNA were extracted using an E.Z.N.A. DNA/RNA isolation kit (Omega Biotek, VWR) 11. Primary human chondrocytes were prepared and cultured as previously reported 12.

The Oxford Musculoskeletal Biobank collection provided samples of OA trapezium. Patients gave their informed consent for sample collection (REC reference no. 09/H0606/11). Trapezium cartilage was dissected from the bone within 2 hours of surgical removal from the joint, and the tissue was then snap frozen in liquid nitrogen and stored at −80°C. Cartilage and bone tissue were ground using a Cryo‐Cup Grinder (Biospec). RNA was extracted from cartilage using an RNeasy Micro kit (Qiagen). DNA was extracted from the bone using DNAzol (Thermo Fisher Scientific).

Further details regarding both the Newcastle and Oxford patients can be found in Supplementary Table 1 (available on the *Arthritis & Rheumatology* web site at http://onlinelibrary.wiley.com/doi/10.1002/art.40545/abstract).

**Quantitation of gene expression.** Synthesis of complementary DNA (cDNA) and analysis by quantitative reverse transcription–polymerase chain reaction (qRT‐PCR) were performed as described previously 9. Predesigned TaqMan assays (Integrated DNA Technologies) were used to quantify expression of the housekeeping genes *HPRT1*,* 18S*, and *GAPDH* and the target genes *COL2A1*,* COL10A1*,* SOX9*,* ACAN*,* ADAMTS5*,* VEGFA*,* RUNX2*, and *MMP13*. For target genes *ALDH1A2*,* RARA*,* RARB*,* RARG*,* RXRA*,* RXRB*,* CRABP2*, and *CYP26B1*, primers were designed using the Roche probe library system (see Supplementary Table 2, available on the *Arthritis & Rheumatology* web site at http://onlinelibrary.wiley.com/doi/10.1002/art.40545/abstract). *P* values were calculated using a Mann‐Whitney 2‐tailed exact test.

**RNA‐sequencing (RNA‐seq) analysis.** RNA‐seq analysis was performed on the cartilage of 10 patients with hip OA and 6 patients with NOF fracture (non‐OA controls) (the RNA‐seq data have been deposited in the NCBI Gene Expression Omnibus [accession no. GSE111358]). Quality of the raw sequencing data was assessed using Fastqc (version 0.11.5) and compiled for experiment‐wide context using MultiQC (version 1.0dev) 13, 14. Salmon software (version 0.8.2) was used to quantify raw Fastq files, based on an index derived from Gencode V24 transcript sequences 15, 16. Salmon was run in sequence and G/C bias correction models, with 100 bootstraps. Abundance estimations were analyzed in R (version 3.4.1), and estimates were imported using Tximport (version 1.4.0) 17, 18, 19. Statistical modeling was performed using DESeq2 (version 1.16.1) 20 to library‐scale normalize the raw counts and fit a negative binomial generalized linear model. Hypothesis testing was performed using the DESeq2 implementation of the Wald test. Statistical significance of the analyzed genes was determined on the basis of a false discovery rate–corrected *P* value of <0.01 and a fold change filter of 2.

**Genotyping.** The rs3204689 SNP was genotyped by a restriction fragment length polymorphism assay. The SNP was amplified by PCR using the primers listed in Supplementary Table 3 (available on the *Arthritis & Rheumatology* web site at http://onlinelibrary.wiley.com/doi/10.1002/art.40545/abstract). The discriminatory enzyme was *SfcI* (New England Biolabs), which cuts at the non‐risk G allele.

The rs4238326 SNP was genotyped by pyrosequencing (see Supplementary Table 3 for the primers used). PCR products were analyzed using the PyroMark Q24 MDx platform (Qiagen), with use of the sequencing primer listed in Supplementary Table 3 and PyroMark Gold Q96 reagents, in accordance with the manufacturer's instructions.

**Determination of allelic expression imbalance (AEI).** AEI at rs3204689 was quantified by pyrosequencing, using the pyrosequencing methods described above and the PCR and sequencing primers listed in Supplementary Table 3. Analysis of the results was performed as described previously 9. *P* values were calculated using a Mann‐Whitney 2‐tailed exact test.

**Determination of mRNA stability.** Chondrocytes were isolated from the cartilage of 4 patients with knee OA who were heterozygous at rs3204689. The cells were seeded in 6‐well plates at 400,000 cells per well, and then treated with 5 μg/ml actinomycin D (Sigma‐Aldrich) for 0, 4, 8, 12, and 24 hours. Nucleic acid was extracted using TRIzol reagent (Life Technologies), and expression of *ALDH1A2* was measured by qRT‐PCR.

AEI at rs3204689 was assessed by pyrosequencing. Expression of *ALDH1A2* mRNA in the combined group of patients was compared at each time point relative to time 0. *P* values were calculated using a Mann‐Whitney 2‐tailed exact test.

**RNA interference (RNAi).** RNAi was performed in chondrocytes isolated from the cartilage of 3 patients with knee OA, essentially as described previously 9. For each patient, the cells were seeded in each well of a 6‐well plate at a density of 350,000 cells per well. Cells were transfected with 50 n*M* Dharmacon ON‐TARGET*plus* SMARTpool small interfering RNA (siRNA) targeted against *ALDH1A2* (L‐008118‐00) or a nontargeting siRNA as the control (D‐001810‐10‐20). RNA and protein were extracted concurrently using the Nucleospin RNA/protein kit (Macherey‐Nagel). Gene expression was assessed by qRT‐PCR using cDNA synthesized from RNA extracted from each well, with 5 technical repeats per analyzed gene. *P* values were calculated using a Student's 2‐tailed *t*‐test.

**Immunoblotting.** For immunoblot analysis of RALDH2 depletion following RNAi, 10 μg of protein was resolved on 10% (weight/volume) sodium dodecyl sulfate–polyacrylamide gel electrophoresis (SDS‐PAGE) gels. Blots were probed with an anti‐ALDH1A2 antibody (HPA010022; Atlas Antibodies) or anti‐GAPDH antibody (Cell Signaling Technology). RALDH2 depletion was quantified using ImageJ software 21, with RALDH2 values normalized to the levels of anti‐GAPDH. *P* values were calculated using Student's 2‐tailed *t*‐test.

For immunoblot analysis of RALDH2 in ground trapezium bone samples, cellular protein was extracted with radioimmunoprecipitation assay buffer. Twenty micrograms of clarified lysate was resolved by SDS‐PAGE and immunoblotted as described above.

**Luciferase reporter assays.** DNA regions surrounding the 39 SNPs selected for analysis were cloned and used for luciferase reporter analysis, essentially as described previously 9. Primer sequences are listed in Supplementary Table 4 (available on the *Arthritis & Rheumatology* web site at http://onlinelibrary.wiley.com/doi/10.1002/art.40545/abstract). The cell lines SW1353 and HEK 293 (both from ATCC) were seeded at 10,000 cells per well in 96‐well plates, and after 24 hours, they were transfected with the firefly and *Renilla* luciferase constructs. *P* values were calculated using a Mann‐Whitney 2‐tailed exact test.

**Electrophoretic mobility shift assays (EMSAs).** Nuclear protein was extracted from SW1353 and HEK 293 cells as previously described 22. For each allele of each of the 8 SNPs studied, forward and reverse single‐stranded DY682‐labeled oligonucleotides (Eurofins MWG Operon), spanning 15 bp each side of the SNP (see Supplementary Table 5, available on the *Arthritis & Rheumatology* web site at http://onlinelibrary.wiley.com/doi/10.1002/art.40545/abstract), were annealed to generate double‐stranded probes. EMSAs were then undertaken as previously described 22. For rs12915901, an Ets random competitor probe was used as a negative control (see Supplementary Table 6, available on the *Arthritis & Rheumatology* web site at http://onlinelibrary.wiley.com/doi/10.1002/art.40545/abstract).

## Results

**Figure 1:**
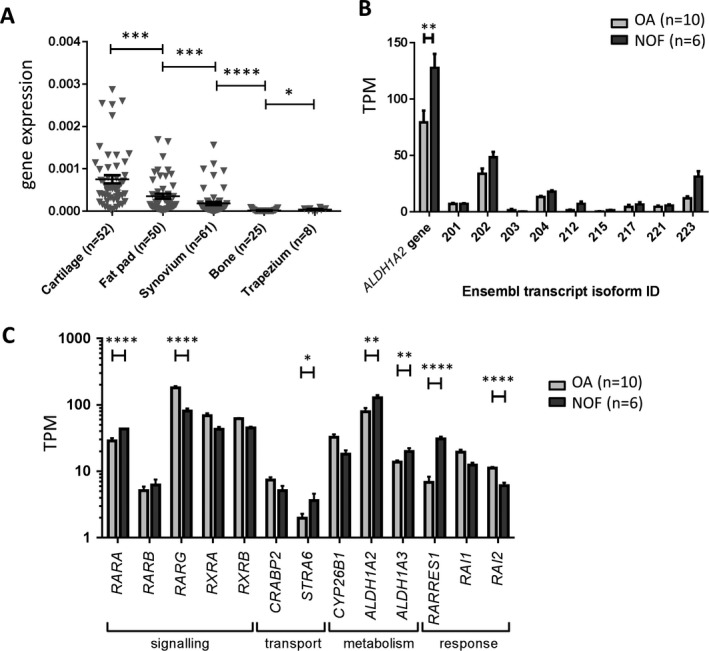
Expression analysis of *ALDH1A2* and retinoic acid pathway genes in multiple tissue samples from patients with osteoarthritis (OA). **A,** Expression of *ALDH1A2* mRNA was measured by quantitative reverse transcription–polymerase chain reaction in OA cartilage, fat pad, synovium, bone, and trapezium. **B,** Expression of *ALDH1A2* gene and transcript isoforms (designated by their Ensembl database identification [ID] numbers) was measured in hip cartilage from OA patients and patients with neck‐of‐femur (NOF) fracture as controls, using RNA‐sequencing data. The category “*ALDH1A2* gene” represents all isoforms combined. **C**, Expression of a panel of retinoic acid pathway genes was measured in hip cartilage from OA patients and non‐OA NOF controls, using RNA‐sequencing data. The data for *ALDH1A2* are replotted for comparison. In **C**, transcripts per million (TPM) kilobase values are plotted as the log^10^. *P* values were calculated using Mann‐Whitney 2‐tailed exact test in **A** and Wald test within the DESeq2 package in **B** and **C**. In **A,** symbols represent individual samples; horizontal lines with bars show the mean ± SEM. In **B** and **C,** results are the mean ± SEM. * = *P* < 0.05; ** = *P* < 0.01; *** = *P* < 0.001; **** = *P* < 0.0001.

**Quantitative expression of** ***ALDH1A2*** **in joint tissues.** We measured *ALDH1A2* expression by qRT‐PCR in the cartilage, fat pad, synovium, and trabecular bone from OA patients who had undergone knee or hip joint replacement surgery. Expression was also measured in osteochondral tissue from patients who had undergone a trapeziectomy. Expression was highest in the cartilage and lowest in the bone and trapezium. In fact, the expression of *ALDH1A2* was below the limit of detection (established as a threshold cycle value of ≥40) in 17 of the 25 bone samples and 2 of the 8 trapezium samples (Figure 1A).

Having confirmed expression of *ALDH1A2* mRNA in the joint tissues, including samples from the trapezium of patients who underwent a trapeziectomy, we next confirmed expression of the RALDH2 protein by immunoblotting of the protein extracted from the trapezium tissue of patients with hand OA (see Supplementary Figure 1, available on the *Arthritis & Rheumatology* web site at http://onlinelibrary.wiley.com/doi/10.1002/art.40545/abstract).

Using RNA‐seq data from the cartilage of OA patients and that from non‐OA controls (i.e., age‐matched patients with NOF fracture) who had undergone hip replacement surgery, we characterized the expression pattern of the *ALDH1A2* transcript isoforms. The Ensembl database (available at http://www.ensembl.org/index.html) lists 25 isoforms for this gene, of which 8 are predicted to be protein coding. Nine of the 25 isoforms were expressed in the analyzed cartilage above a mean transcripts‐per‐million threshold of 1, including 4 of the 8 protein‐coding isoforms (Figure 1B) (see also Supplementary Table 7, available on the *Arthritis & Rheumatology* web site at http://onlinelibrary.wiley.com/doi/10.1002/art.40545/abstract). When assessing expression of all *ALDH1A2* isoforms combined (designated the *ALDH1A2* gene in Figure 1B), there was a 0.3‐fold decrease in *ALDH1A2* expression in the OA cartilage compared to the non‐OA control cartilage (*P* < 0.01).

**Differential expression of RA pathway genes in OA cartilage.** The reduced expression of *ALDH1A2* in OA compared to non‐OA cartilage prompted us to investigate our RNA‐seq data for the relative expression of other genes active in the retinoic acid pathway (see Supplementary Table 8, available on the *Arthritis & Rheumatology* web site at http://onlinelibrary.wiley.com/doi/10.1002/art.40545/abstract). Of a panel of 13 genes selected as a representative cross‐section of the retinoic acid pathway, and due to their relatively abundant levels of expression, several of these genes were differentially expressed between OA cartilage and non‐OA control cartilage (Figure 1C). *RARRES1*, which codes for retinoic acid receptor responder 1, was one of the most significantly differentially expressed genes, with a 2.4‐fold decreased expression in OA cartilage (*P* = 6 × 10^−12^). The results of this analysis imply that there is a widespread differential regulation of the retinoic acid pathway between OA and non‐OA cartilage.

**Figure 2:**
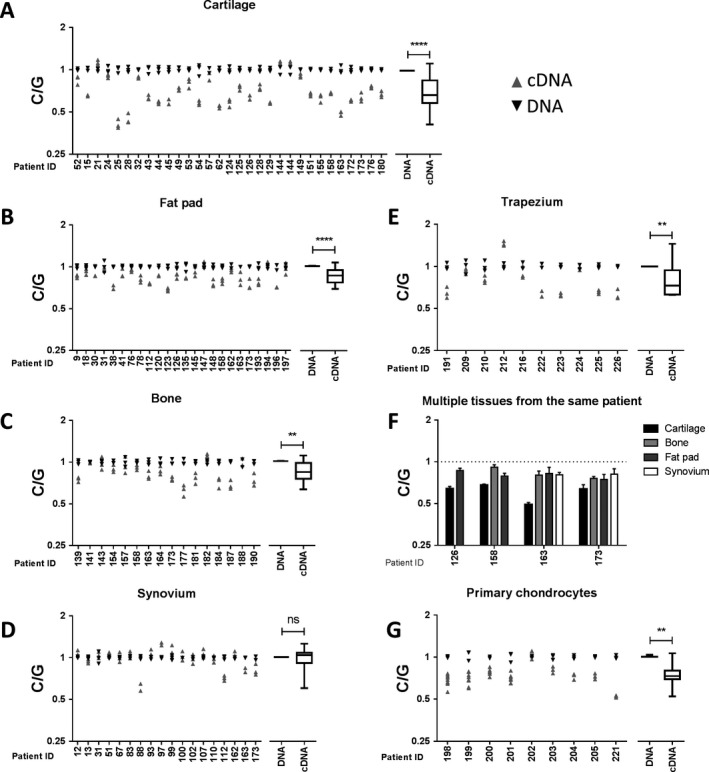
Allelic expression imbalance (AEI) analysis of *ALDH1A2*. **A–E** and **G**, AEI analysis of rs3204689 was carried out in osteoarthritis (OA) patient cartilage (n = 31) (**A**), fat pad (n = 24) (**B**), bone (n = 16) (**C**), synovium (n = 18) (**D**), trapezium (n = 10) (**E**), and primary chondrocytes (n = 9) (**G**). Plots in the left panels show the risk/non‐risk (C/G) allelic ratios, with a ratio of <1 indicating decreased expression of the C allele. The right panels show the mean values for DNA and cDNA from all patients combined, with results represented as box‐and‐whisker plots, in which the lines within the box represent the median, the box represents the 25th to 75th percentiles, and the whiskers represent the minimum and maximum values. A minimum of 3 technical repeats were performed for each patient's DNA and cDNA. *P* values were calculated using Mann‐Whitney 2‐tailed exact test. **F,** AEI analysis was carried out in 4 OA patients for whom cartilage samples and tissue from at least one other site were available. The broken horizontal line indicates a C/G ratio of 1, which is indicative of no allelic imbalance. Values are the mean ± SD AEI plotted for each individual in each tissue tested. Individual patients are designated by their anonymized identification (ID) numbers. ** = *P* < 0.01; **** = *P* < 0.0001. NS = not significant.

**AEI analysis of** ***ALDH1A2*** **.** The investigators who reported the association of OA risk with *ALDH1A2* observed a reduction in expression of the OA risk–conferring allele of the gene in AEI analysis. We replicated this observation, detecting an average reduction of 28% in the expression of the risk C allele in the cartilage of patients who were heterozygous at rs3204689 (*P* < 0.0001), with the majority of patients demonstrating AEI (Figure 2A).

In our expanded analysis, we observed an average reduction of 14% in the expression of the risk C allele in the fat pad (*P* < 0.0001) (Figure 2B) and a reduction of 15% in the bone samples (*P* = 0.001) (Figure 2C), whereas in the synovium, the reduction was nonsignificant (average reduction 2%; *P* > 0.05) (Figure 2D). We observed an average reduction of 18% in the expression of the risk allele in the trapezium (*P* < 0.01) (Figure 2E). Despite the nonsignificant AEI observed in synovium samples, there were individuals who did demonstrate clear AEI in the synovium (patients 88 and 112 in Figure 2D).

The reduction in expression of the OA risk allele was less profound in these noncartilaginous joint tissues, as was most clearly demonstrated when we compared AEI ratios in 4 patients for whom cartilage tissue and tissue from at least 1 other site from the same joint could be concurrently analyzed. In each patient, cartilage showed a larger AEI than that observed in any of the noncartilaginous tissue samples (Figure 2F).

It is noteworthy that in a small number of patients, the OA risk–conferring C allele was expressed at a higher level, rather than a lower level, than was the non‐risk allele (Figures 2A–E). This was most obvious in the synovium of patient 97 (Figure 2D) and the osteochondral trapezium of patient 212 (Figure 2E).

Finally, we assessed AEI stability during tissue culture by extracting chondrocytes from the knee cartilage of 9 OA patients who were heterozygous at rs3204689, culturing the cells in monolayer for a minimum of 10 days and then undertaking AEI analysis (Figure 2G). We observed an average reduction of 25% in the expression of the risk allele (*P* = 0.004), which is comparable to the reduction seen in OA cartilage tissue samples (Figure 2A), thus suggesting that the AEI is stable during cell division in vitro.

**Figure 3:**
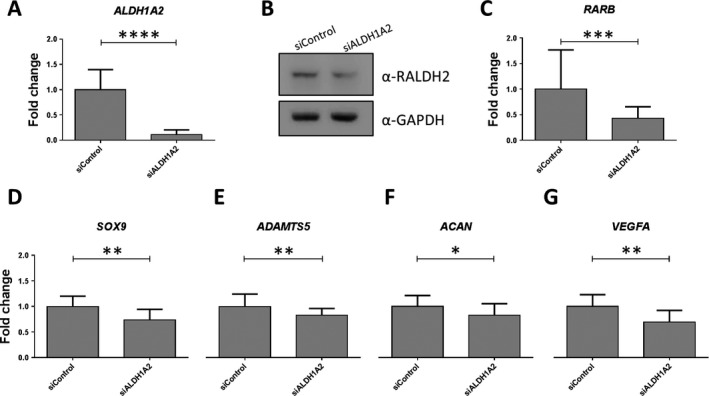
Effects of knockdown of *ALDH1A2* in primary chondrocytes from patients with osteoarthritis (OA). **A,** *ALDH1A2* knockdown with small interfering RNA (siRNA) targeting *ALDH1A2* (siALDH1A2) was carried out in cultured knee chondrocytes from 3 OA patients, in comparison to the effects of a nontargeting siRNA control (siControl). *ALDH1A2* expression was measured by quantitative reverse transcription–polymerase chain reaction. Values represent the mean ± SD fold change in expression compared to siControl, with the data combined for the 3 patients. **B,** Representative results from immunoblotting demonstrate depletion of RALDH2 protein following *ALDH1A2* knockdown. GAPDH was used as a loading control. **C**–**G**, The fold change in expression of *RARB* **(C)**,*SOX9* **(D)**,*ADAMTS5* **(E)**,*ACAN* **(F)**, and *VEGFA* **(G)** following *ALDH1A2* knockdown, relative to that with the siControl, was assessed in chondrocytes from the 3 OA patients combined. Values are the mean ± SD. *P* values were calculated using Student's 2‐tailed *t*‐test. * = *P* < 0.05; ** = *P* < 0.01; *** = *P* < 0.001; **** = *P* < 0.0001.

**Effect of knockdown of** ***ALDH1A2*** **on chondrocyte gene expression.** Having demonstrated that the OA risk–conferring C allele of rs3204689 is correlated with decreased expression of *ALDH1A2*, we next modeled this effect. Chondrocytes were isolated from the knee cartilage of 3 OA patients, and then cultured in monolayer and subjected to *ALDH1A2* knockdown by RNAi. Compared to the effects of the scrambled siRNA control, siRNA targeting *ALDH1A2* achieved a mean knockdown at the *ALDH1A2* mRNA level of 89% (*P* < 0.0001) (Figure 3A), with a 22% reduction at the protein level (*P* < 0.05) (Figure 3B).

We next assessed the effect of this knockdown of *ALDH1A2* on the expression of 7 retinoic acid pathway genes (*RARA*,* RARB*,* RARG*,* RXRA*,* RXRB*,* CRABP2*, and *CYP26B1*) and 8 chondrogenic genes (*SOX9*,* ADAMTS5*,* MMP13, ACAN*,* COL2A1*,* COL10A1*,* RUNX2*, and *VEGFA*). Depletion of *ALDH1A2* correlated with a significant reduction in the expression of *RARB* (*P* = 0.001), *SOX9* (*P* = 0.002), *ADAMTS5* (*P* = 0.007), *ACAN* (*P* = 0.04), and *VEGFA* (*P* = 0.004) (Figures 3C–G). Therefore, in this model system, depletion of *ALDH1A2* transcript and of its protein in OA knee cartilage had a significant impact on genes that encode regulators of cartilage homeostasis.

**Lack of involvement of UTR SNPs in regulating transcript stability.** The 3′‐UTRs offer binding sites for microRNAs (miRNAs) that can regulate transcript stability. The 3′‐UTR of *ALDH1A2* contains 2 SNPs that correlate with the rs3204689 association signal: rs3204689 itself and rs9325 (r^2^ = 0.96 with rs3204689). A search of TargetScan ( http://www.targetscan.org/vert_71/) revealed that both of these SNPs reside within predicted miRNA binding sites. In order to assess whether the eQTL operating on *ALDH1A2* was the result of miRNA‐mediated transcript degradation, we measured AEI in cultured chondrocytes following treatment with the transcriptional inhibitor actinomycin D. If the ratio of AEI endures following treatment, this would imply that the AEI results from differential transcription between alleles, rather than differential transcript stability.

**Figure 4:**
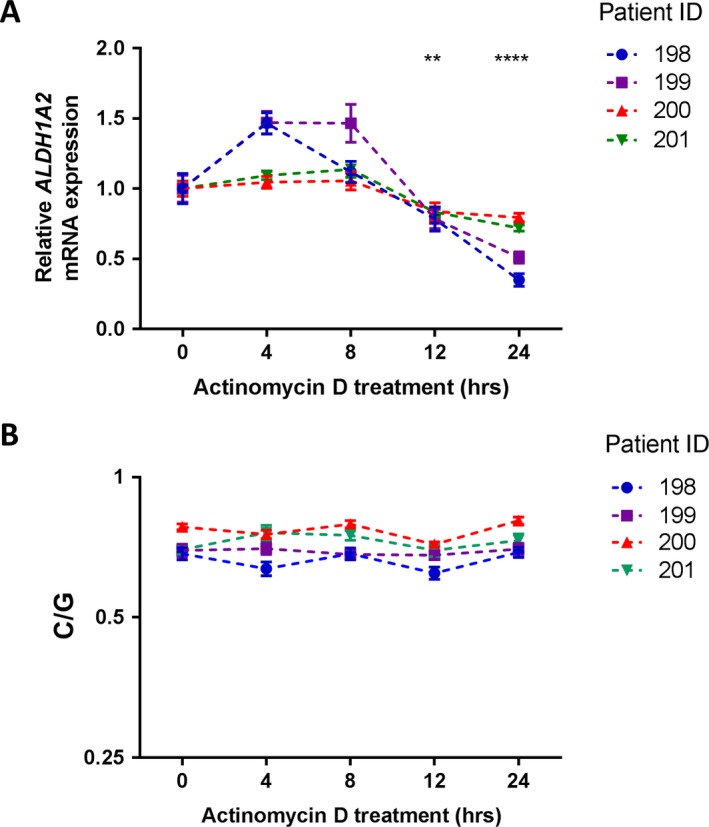
*ALDH1A2* transcript stability in osteoarthritis (OA) chondrocytes. **A,** Chondrocytes from 4 OA patients were cultured in the presence of actinomycin D for 0, 4, 8, 12, or 24 hours and the effects on *ALDH1A2* mRNA expression were assessed by quantitative reverse transcription–polymerase chain reaction. At each time point, the mean ± SEM *ALDH1A2* mRNA expression level in the 4 patients combined was compared to time 0. *P* values were calculated using a Mann‐Whitney 2‐tailed exact test. **B,** The allelic ratio (C/G) at rs3204689 was determined by pyrosequencing in the chondrocytes from the 4 OA patients at each time point. Values are the mean ratio determined from 6 technical repeats. Individual patients are designated by their anonymized identification (ID) numbers. ** = *P* < 0.01; **** = *P* < 0.0001.

We investigated chondrocytes from 4 patients with knee OA who were heterozygous at rs3204689. Levels of *ALDH1A2* mRNA expression were significantly decreased in the chondrocytes after 12 hours (*P* < 0.01) and 24 hours (*P* < 0.0001) of actinomycin D treatment, compared to time 0 (Figure 4A), but the allelic ratio remained stable for the duration of the time course in each patient's cells (Figure 4B). These data therefore support the notion that an effect on transcription, rather than transcript stability, is the mechanism through which the association signal impacts on *ALDH1A2* expression.

**Triage to identify functional candidate SNPs.** We aimed to identify SNPs that correlated with the OA association signal, and in which the 2 SNP alleles demonstrated differential transcriptional activity. Such SNPs would be strong functional candidates responsible for the AEI. To achieve this, we applied a triage system that had 3 sequential stages: 1) identification of SNPs showing a correlation (r^2^ ≥ 0.8) with either rs3204689 or rs4238326 and that could be predicted in silico to be potentially functional; 2) luciferase reporter analysis of both alleles of these SNPs in transformed cell lines; and 3) further functional characterization by EMSA of the SNPs that demonstrated AEI in the luciferase analysis.

**SNPs correlated with the OA association signal.** Using HaploReg version 4.1 ( http://archive.broadinstitute.org/mammals/haploreg/haploreg.php), we identified 54 SNPs that strongly correlated with rs3204689 and 8 SNPs that strongly correlated with rs4238326 (each r^2^ ≥ 0.8) (see Supplementary Tables 9 and 10, available on the *Arthritis & Rheumatology* web site at http://onlinelibrary.wiley.com/doi/10.1002/art.40545/abstract). All resided within the gene body or immediately downstream of *ALDH1A2*, and encompassed a 145‐kb region.

We screened all 62 of these SNPs, as well as rs3204689 and rs4238326, for predicted transcriptional functionality using RegulomeDB ( http://www.regulomedb.org) (for more details on the RegulomeDB scores, see Supplementary Figure 2, available on the *Arthritis & Rheumatology* web site at http://onlinelibrary.wiley.com/doi/10.1002/art.40545/abstract). This analysis identified 38 positive SNPs (as indicated by the RegulomeDB scores listed in Supplementary Tables 9 and 10), and these were taken forward for luciferase analysis. We also included rs4646563, despite there being no supporting RegulomeDB data for this SNP, as it resides close to the CpG dinucleotide cg12031962, which marks an *ALDH1A2* methylation QTL (mQTL) that we described in a previous report 23. In total, we took forward 39 SNPs for luciferase analysis.

**Allelic expression differences identified at 8 SNPs by luciferase analysis.** For these 39 SNPs, each allele was cloned into a pGL3‐promoter plasmid (with multiple SNPs cloned together if they were <200 bp apart) and relative luciferase activity was compared in 2 human cell lines, SW1353 chondrosarcoma cells and HEK 293 cells. The former was chosen because of its chondrocyte origin, and the latter because it abundantly expresses *ALDH1A2*.

**Figure 5:**
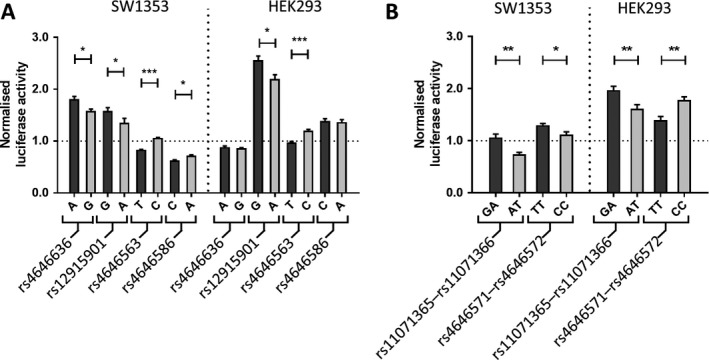
Luciferase reporter analysis of single‐nucleotide polymorphism (SNP) alleles. Four SNPs correlating with rs3204689 (**A)** and 2 SNP pairs correlating with rs4238326 (**B**) demonstrated allelic expression differences in SW1353 and HEK 293 cells. Values are plotted as the mean ± SEM normalized luciferase activity from 6 biologic repeats, each with 6 technical repeats. For each SNP, the osteoarthritis risk–associated allele is shown as light gray–shaded bars. The horizontal dashed line indicates no difference in expression relative to the *Renilla* transfection control (normalized luciferase activity value of 1.0). *P* values were calculated using a Mann‐Whitney 2‐tailed exact test. * = *P* < 0.05; ** = *P* < 0.01; *** = *P* < 0.001.

Six constructs encompassing a total of 8 SNPs displayed significant allelic differences in transcriptional activity (*P* < 0.05) (Figures 5A and B). These SNPs were rs4646636, rs12915901, rs4646563, and rs4646586, which correlated with rs3204689, and rs11071365, rs11071366, rs4646571, and rs4646572, which correlated with rs4238326.

Of the 8 SNPs, rs12915901 was particularly noteworthy in that both the A and G alleles of this SNP acted as an enhancer in both cell types (normalized luciferase activity >1.0), with the A allele (equivalent to the risk‐conferring C allele of rs3204689) showing lower expression relative to the non‐risk G allele in both cell types. The relative reduction in expression of the risk allele observed for rs12915901 was 15% in SW1353 cells and 14% in HEK 293 cells. These reductions are comparable to those observed in OA patient cartilage (28%), fat pad (14%), and bone (15%) for the C allele of rs3204689 (Figure 2). As such, rs12915901 recapitulated the AEI seen in OA patients. The additional 7 SNPs that displayed allelic differences in transcriptional activity did not meet the same criteria as met by rs12915901.

**Figure 6:**
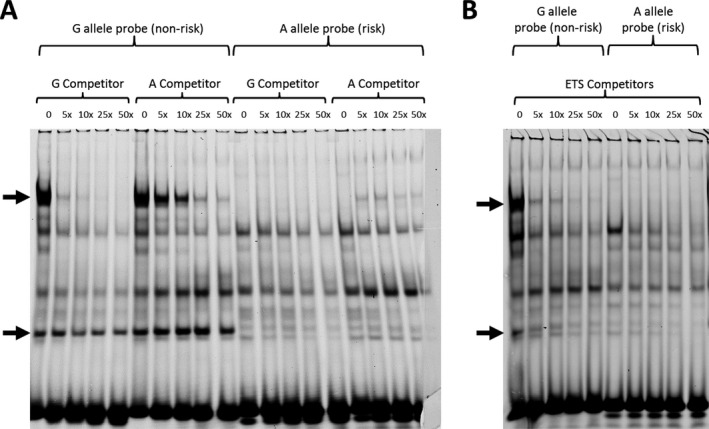
Electrophoretic mobility shift assay (EMSA) analyses of expression of the rs12915901 G and A alleles in SW1353 cells. **A,** Increasing concentrations of unlabeled G allele and the A allele competitor were added to the EMSA reactions containing cell nuclear extract and a G or A allele probe. **B,** Increasing concentrations of an unlabeled Ets competitor were added to the EMSA reaction containing a G allele or an A allele probe. **Arrows** indicate 2 complexes binding with much greater affinity to the G allele than to the A allele.

**EMSA characterization of differential allelic binding at rs12915901.** We used EMSAs to characterize protein complex binding to each of the 8 positive SNPs that emerged from our luciferase analysis. This analysis revealed differential allelic binding of 2 protein complexes to rs12915901 (results in SW1353 cells in Figure 6A; results in HEK 293 cells in Supplementary Figure 3A, available on the *Arthritis & Rheumatology* web site at http://onlinelibrary.wiley.com/doi/10.1002/art.40545/abstract). We found no consistent allelic differences in protein complex binding for the other 7 SNPs analyzed. The 2 rs12915901 complexes bound almost exclusively to the non‐risk G allele of the SNP, with the higher molecular weight complex competing much less efficiently with the A allele than with the G allele competitor (Figure 6B).

A search of the online databases JASPAR ( http://jaspar.genereg.net) and UniProbe ( http://the_brain.bwh.harvard.edu/uniprobe/) identified the Ets family of transcription factors as potentially binding to rs12915901 and its flanking sequence, but only for the non‐risk G allele: the highly conserved Ets DNA binding domain is 5′‐GGAA‐3′, which is intact for the G allele of rs12915901 but is abolished in the A allele, with the sequence changing to 5′‐GAAA‐3′ (underline indicates the rs12915901 base).

This hypothetical loss of Ets binding was consistent with our EMSA data, in that the 2 protein complexes bind to the G allele but not the A allele of the SNP. We therefore used an Ets competitor sequence in our EMSA. This competed very efficiently for binding to the 2 protein complexes, implying that they do contain at least 1 Ets transcription factor (Figure 6B and Supplementary Figure 3B, http://onlinelibrary.wiley.com/doi/10.1002/art.40545/abstract). A random competitor sequence had no effect (data not shown). Ets is one of the largest family of transcription factors, with 29 Ets genes in humans. Using our RNA‐seq data, we determined that the majority of these Ets transcription factors were expressed in cartilage.

## Discussion

Retinoic acid plays a crucial role in organism development 24, via its regulatory effects on chondrogenesis and osteogenesis 25. Its synthesis involves the oxidation of retinaldehyde to retinoic acid. This step is catalyzed by the RALDH enzymes, of which there are 3 in humans (RALDH1, RALDH2, and RALDH3), each coded for by a separate gene (*ALDH1A1*,* ALDH1A2*, and *ALDH1A3*). RALDH2/*ALDH1A2* is pivotal, with knockout of the mouse ortholog being lethal 24.

A 2014 report discussing an association with OA of SNPs at the *ALDH1A2* locus 6 was the first occasion in which the retinoic acid pathway had been associated with OA at a genome‐wide significance level, and those findings highlighted the fact that a molecule key to early postnatal development can have an impact on a disease that tends to develop in older individuals. That prior report contained a number of features common in OA genetic studies: 1) the signal is to a gene involved in a regulatory pathway; 2) the functional effect of the genetic susceptibility is on gene expression; and 3) the signal is not a risk factor for disease at all skeletal sites examined 2, 4, 5.

We set out to replicate and expand upon the findings included in the 2014 study. We confirmed that the risk‐conferring C allele of rs3204689 correlated with reduced expression of *ALDH1A2* in cartilage, and demonstrated that this effect was common in the fat pad and bone of OA patients, albeit to a lower degree. AEI was less common in the synovium examined. Our data suggest that the functional consequence of the genetic risk is not uniform across joint tissues, with cartilage being the major target tissue. The fact that cartilage showed a more pronounced AEI when we studied several joint tissue sites from the same individual suggests that nongenetic modulators have a role. We have previously demonstrated that *ALDH1A2* is subject to an mQTL that correlates with the association signal 23. A detailed analysis of DNA CpG methylation and *ALDH1A2* AEI in multiple joint tissues from the same donor is clearly merited.

We examined osteochondral tissue from patients with hand OA who had undergone a trapeziectomy. Severe thumb OA was one of the clinical phenotypes for which an association with *ALDH1A2* was demonstrated in the 2014 report. In the present study, we observed statistically significant AEI in the osteochondral tissue, confirming, for the first time, that the risk allele at this locus correlates with decreased expression of *ALDH1A2* in the trapezium of patients with hand OA.

Our RNA‐seq data revealed reduced expression of *ALDH1A2* in OA hip cartilage compared to non‐OA hip cartilage. Reduction in expression in OA cartilage, combined with carriage of 1 or 2 copies of the low‐expressing C allele of rs3204689, may be the risk‐conferring scenario. In the same RNA‐seq data set, we observed differential expression of several retinoic acid–related genes. This implies that a systemic alteration in the activity of the retinoic acid pathway occurs in OA. We are not aware of any reports that have discussed a significant association of any of the other investigated retinoic acid genes with OA, but an analysis of these genes as candidate genes may be worthwhile.

A functional role of retinoic acid and retinoic acid receptors in the pathogenesis of OA has been described. Increased concentrations of retinoic acid receptor ligands were previously reported in synovial fluid samples extracted from patients with OA as compared to non‐OA controls 26. These increased concentrations of retinoic acid metabolites and derivatives are suggested to be detrimental to cartilage through the stimulation of catabolic processes in chondrocyte explant cultures. Paradoxically, our data suggest that decreased *ALDH1A2* expression, and, by extrapolation, decreased retinoic acid availability, may also compromise cartilage integrity through modulation of *SOX9* expression. The catabolic effects of altered retinoic acid levels in cartilage perhaps suggest that the tight regulation of retinoic acid production during development is also critical for the long‐term maintenance of healthy cartilage.

The results of our studies utilizing treatment of chondrocytes with actinomycin D suggest that the alterations in allelic expression occur as a result of the effects on the rate of transcription rather than on transcript stability, while our knockdown of *ALDH1A2* resulted in the reduced expression of several genes, including the gene coding for the key chondrogenic transcription factor *SOX9*. A recent report highlighted the finding that *ALDH1A2* expression is a positive determinant of *SOX9* expression in chondrocytes 27, thereby supporting our own observations.

Our in silico and in vitro analyses of SNPs correlating with the OA association signal identified the *ALDH1A2* intronic SNP rs12915901 as a SNP exhibiting differences in allelic activity that matched the allelic effects seen in our patient‐based studies. Our EMSA analyses implicated the Ets family of transcription factors as positive regulators of expression. Members of this family have previously been reported to have a role in OA via regulation of the expression of chondrogenic genes 28, 29. Detailed analysis of these Ets transcription factors in the context of the *ALDH1A2* association signal and *ALDH1A2* expression is now warranted.

We analyzed several samples of OA trapezieum tissue, but the vast majority of our tissue came from hip or knee arthroplasties, reflecting the small volume of hand OA surgical procedures undertaken relative to hip and knee procedures. In our quantification of *ALDH1A2* expression, the trapezium samples displayed relatively low levels compared to the hip and knee cartilage samples. We posit therefore that where the expression of the gene is already low, as in the hand, that joint may not be able to tolerate further reduction in expression brought about by carriage of the risk allele.

In conclusion, we have characterized the eQTL operating on *ALDH1A2* in multiple joint tissue sites, including trapezium samples obtained from patients with hand OA. We highlight the functional effect of decreased *ALDH1A2* expression in human chondrocytes and show that retinoic acid–related genes are differentially expressed between OA diseased cartilage and non‐OA control cartilage. Our findings prioritize a SNP as a functional variant potentially responsible for the modulation of *ALDH1A2* expression. Experiments building on our findings can now be planned to develop strategies to mitigate the effect of the risk of OA conferred by *ALDH1A2*.

## Author Contributions

All authors were involved in drafting the article or revising it critically for important intellectual content, and all authors approved the final version to be published. Drs. Shepherd and Loughlin had full access to all of the data in the study and take responsibility for the integrity of the data and the accuracy of the data analysis.

### Study conception and design

Shepherd, D. Zhu, Reynard, Loughlin.

### Acquisition of data

Shepherd, D. Zhu, Skelton, Combe, Threadgold, L. Zhu, Vincent, Stuart.

### Analysis and interpretation of data

Shepherd, D. Zhu, Skelton, Reynard, Loughlin.

## Supporting information

Supplementary Figure 1

Supplementary Figure 2

Supplementary Figure 3

Supplementary Table 1

Supplementary Table 2

Supplementary Table 3

Supplementary Table 4

Supplementary Table 5

Supplementary Table 6

Supplementary Table 7

Supplementary Table 8

Supplementary Table 9

Supplementary Table 10
