# Supplementary figures and images for: Functional Characterization of the Osteoarthritis Genetic Risk Residing at ALDH1A2 Identifies rs12915901 as a Key Target Variant
Source: Arthritis Rheumatol. 2018 Aug 23;70(10):1577–87. doi: 10.1002/art.40545 (PMC6175168; doi:10.1002/art.40545)

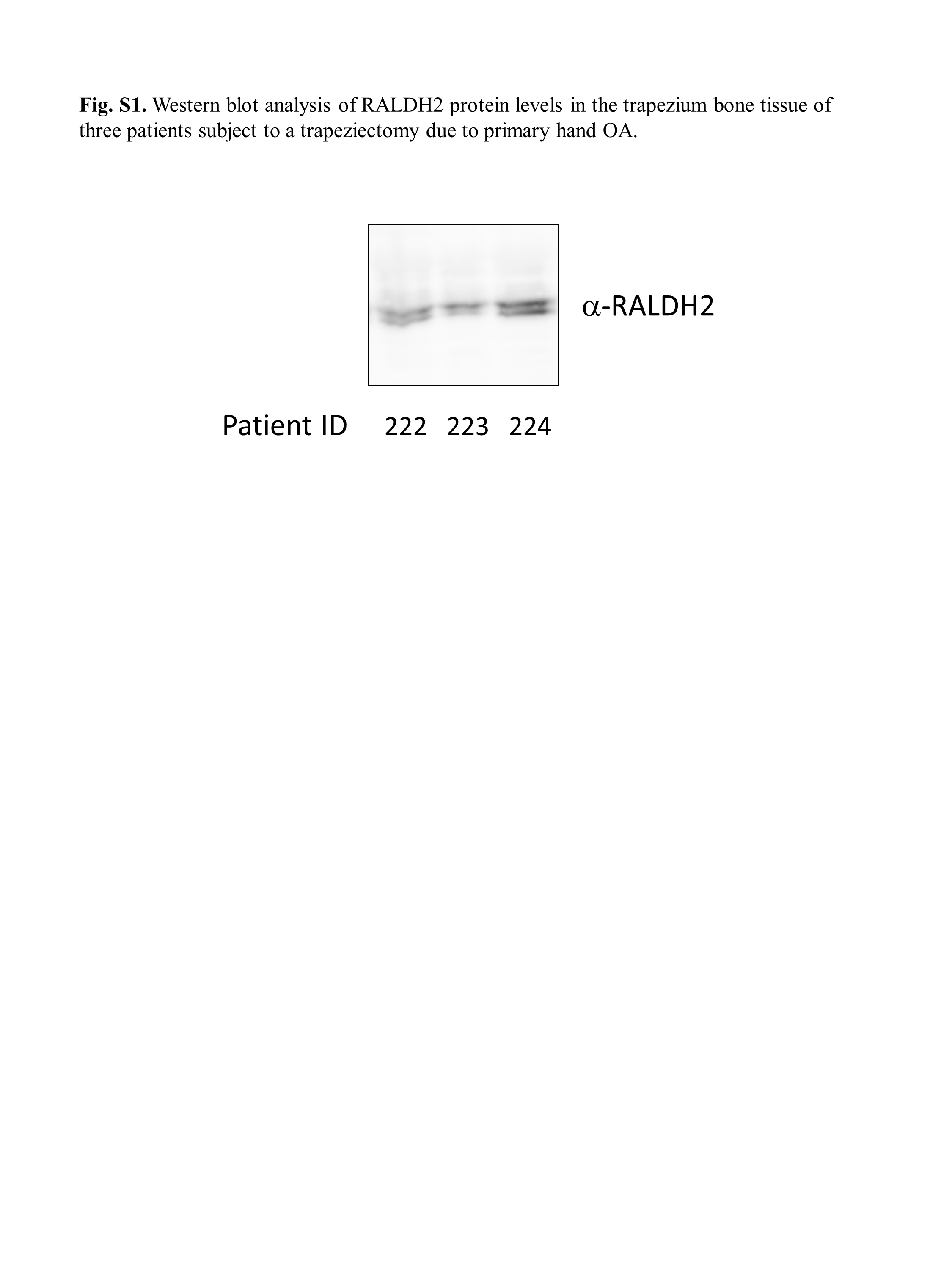

Supplement: Supplementary file 1 — Supplementary Figure 1 [file ART-70-1577-s001.tif]

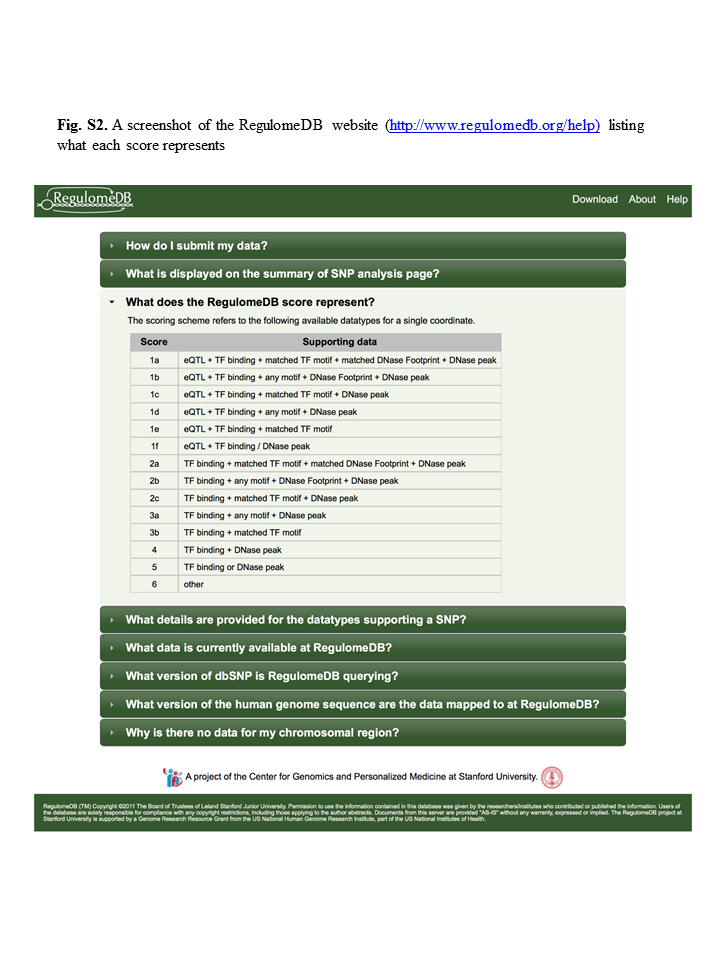

Supplement: Supplementary file 2 — Supplementary Figure 2 [file ART-70-1577-s002.tif]

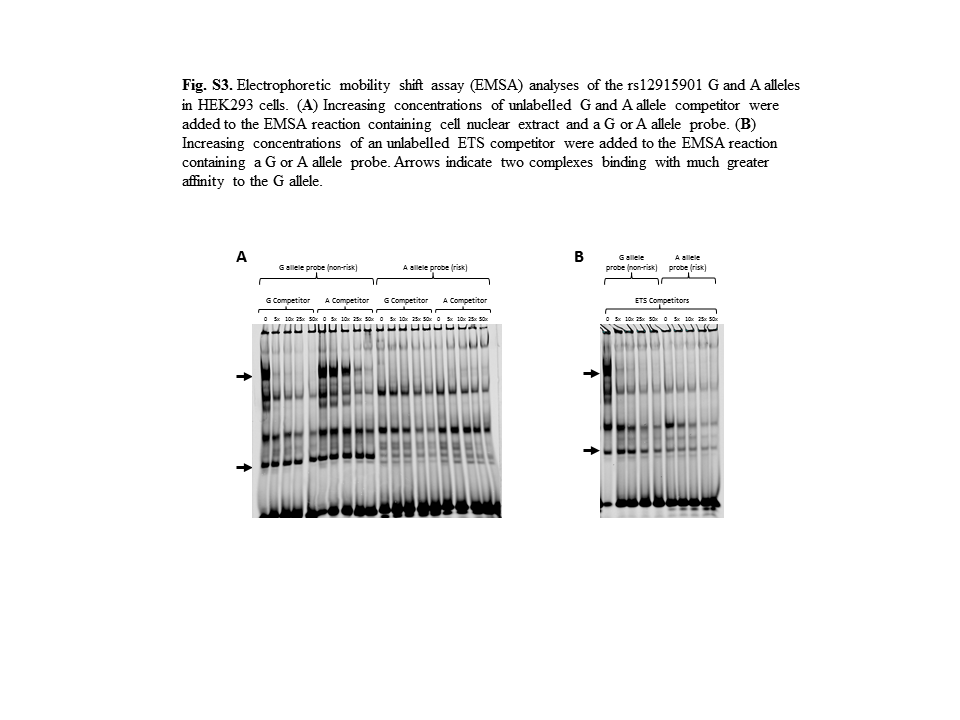

Supplement: Supplementary file 3 — Supplementary Figure 3 [file ART-70-1577-s003.tif]
