# Supplementary material for: Functional Characterization of the Osteoarthritis Genetic Risk Residing at ALDH1A2 Identifies rs12915901 as a Key Target Variant
Source: Arthritis Rheumatol. 2018 Aug 23;70(10):1577–87. doi: 10.1002/art.40545 (PMC6175168; doi:10.1002/art.40545)
Supplement: Supplementary file 4 — Supplementary Table 1 [file ART-70-1577-s004.pdf]

Table S1. The patients used in this study

Details of the patients used for nucleic acid extraction for qRT-PCR and AEI experiments

Patient number	Phenotype	Tissue	Joint site affected	Collection site	Age at surgery (years)	Sex
1	OA	Fat pad & Synovium	knee	Newcastle	55	F
2	OA	Fat pad	knee	Newcastle	68	F
3	OA	Fat pad	knee	Newcastle	63	M
4	OA	Fat pad	knee	Newcastle	74	F
5	OA	Synovium	knee	Newcastle	64	F
6	OA	Synovium	knee	Newcastle	79	F
7	OA	Fat pad	knee	Newcastle	64	M
8	OA	Synovium	knee	Newcastle	73	F
9	OA	Cartilage & Fat pad	knee	Newcastle	69	F
10	OA	Cartilage & Fat pad	knee	Newcastle	57	M
11	OA	Fat pad	knee	Newcastle	64	M
12	OA	Synovium	knee	Newcastle	79	F
13	OA	Synovium	knee	Newcastle	54	M
14	OA	Fat pad	knee	Newcastle	71	F
15	OA	Cartilage	knee	Newcastle	67	M
16	OA	Cartilage	knee	Newcastle	67	F
17	OA	Synovium	knee	Newcastle	67	F
18	OA	Fat pad	knee	Newcastle	72	M
19	OA	Synovium	knee	Newcastle	60	F
20	OA	Fat pad	knee	Newcastle	78	M
21	OA	Cartilage	knee	Newcastle	53	M
22	OA	Cartilage & Fat pad & Synovium	knee	Newcastle	85	M
23	OA	Fat pad	knee	Newcastle	70	M
24	OA	Cartilage	knee	Newcastle	79	F
25	OA	Cartilage	knee	Newcastle	49	M
26	OA	Cartilage	knee	Newcastle	65	M
27	OA	Synovium	knee	Newcastle	72	F
28	OA	Cartilage	knee	Newcastle	85	M
29	OA	Cartilage	knee	Newcastle	72	F
30	OA	Fat pad	knee	Newcastle	70	F
31	OA	Fat pad & Synovium	knee	Newcastle	55	F
32	OA	Cartilage	knee	Newcastle	63	F
33	OA	Fat pad	knee	Newcastle	70	F
34	OA	Cartilage	knee	Newcastle	77	F
35	OA	Cartilage	knee	Newcastle	76	M
36	OA	Fat pad	knee	Newcastle	57	M
37	OA	Fat pad	knee	Newcastle	77	F
38	OA	Fat pad	knee	Newcastle	69	M
39	OA	Fat pad	knee	Newcastle	65	F
40	OA	Fat pad	knee	Newcastle	67	M
41	OA	Fat pad	knee	Newcastle	82	F
42	OA	Cartilage	knee	Newcastle	61	F
43	OA	Cartilage	knee	Newcastle	76	F
44	OA	Cartilage	knee	Newcastle	78	F
45	OA	Cartilage	knee	Newcastle	58	M
46	OA	Cartilage	hip	Newcastle	45	F
47	OA	Cartilage	hip	Newcastle	45	F
48	OA	Cartilage	knee	Newcastle	60	F
49	OA	Cartilage	knee	Newcastle	57	F
50	OA	Cartilage	hip	Newcastle	38	F
51	OA	Synovium	knee	Newcastle	68	F
52	OA	Cartilage	knee	Newcastle	57	F
53	OA	Cartilage	knee	Newcastle	61	F
54	OA	Cartilage	knee	Newcastle	55	M
55	OA	Synovium	knee	Newcastle	50	M
56	OA	Cartilage	knee	Newcastle	82	M
57	OA	Cartilage	hip	Newcastle	66	F
58	OA	Cartilage	knee	Newcastle	76	F
59	OA	Cartilage	hip	Newcastle	89	F
60	OA	Cartilage	knee	Newcastle	64	F
61	OA	Cartilage	hip	Newcastle	59	F
62	OA	Cartilage	knee	Newcastle	68	F

63	OA	Synovium	knee	Newcastle	62	F
64	OA	Synovium	knee	Newcastle	62	F
65	OA	Synovium	knee	Newcastle	68	F
66	OA	Synovium	knee	Newcastle	59	F
67	OA	Synovium	knee	Newcastle	77	M
68	OA	Synovium	knee	Newcastle	75	F
69	OA	Synovium	knee	Newcastle	87	M
70	OA	Synovium	knee	Newcastle	80	M
71	OA	Synovium	knee	Newcastle	65	M
72	OA	Fat pad & Synovium	knee	Newcastle	72	M
73	OA	Synovium	knee	Newcastle	51	F
74	OA	Synovium	knee	Newcastle	62	F
75	OA	Synovium	knee	Newcastle	67	M
76	OA	Fat pad	knee	Newcastle	72	F
77	OA	Synovium	knee	Newcastle	67	M
78	OA	Fat pad	knee	Newcastle	61	M
79	OA	Synovium	knee	Newcastle	68	M
80	OA	Synovium	knee	Newcastle	71	F
81	OA	Synovium	knee	Newcastle	62	M
82	OA	Synovium	knee	Newcastle	66	F
83	OA	Synovium	knee	Newcastle	53	F
84	OA	Synovium	knee	Newcastle	53	M
85	OA	Synovium	knee	Newcastle	57	M
86	OA	Synovium	knee	Newcastle	49	M
87	OA	Synovium	knee	Newcastle	54	F
88	OA	Synovium	knee	Newcastle	75	F
89	OA	Synovium	knee	Newcastle	71	F
90	OA	Fat pad	knee	Newcastle	73	M
91	OA	Synovium	knee	Newcastle	64	M
92	OA	Fat pad	knee	Newcastle	73	F
93	OA	Synovium	knee	Newcastle	63	F
94	OA	Fat pad	knee	Newcastle	72	F
95	OA	Fat pad	knee	Newcastle	61	M
96	OA	Synovium	knee	Newcastle	77	F
97	OA	Synovium	knee	Newcastle	57	M
98	OA	Synovium	knee	Newcastle	60	M
99	OA	Synovium	knee	Newcastle	61	F
100	OA	Synovium	knee	Newcastle	52	F
101	OA	Synovium	knee	Newcastle	76	M
102	OA	Synovium	knee	Newcastle	76	M
103	OA	Synovium	knee	Newcastle	66	F
104	OA	Synovium	knee	Newcastle	85	M
105	OA	Synovium	knee	Newcastle	73	F
106	OA	Synovium	knee	Newcastle	60	M
107	OA	Synovium	knee	Newcastle	72	F
108	OA	Synovium	knee	Newcastle	70	F
109	OA	Synovium	knee	Newcastle	51	F
110	OA	Synovium	knee	Newcastle	66	F
111	OA	Synovium	knee	Newcastle	81	F
112	OA	Fat pad & Synovium	knee	Newcastle	81	F
113	OA	Fat pad	knee	Newcastle	71	F
114	OA	Fat pad	knee	Newcastle	78	M
115	OA	Fat pad	knee	Newcastle	67	F
116	OA	Synovium	knee	Newcastle	68	M
117	OA	Synovium	knee	Newcastle	58	F
118	OA	Fat pad & Synovium	knee	Newcastle	87	F
119	OA	Cartilage	knee	Newcastle	54	F
120	OA	Fat pad	knee	Newcastle	79	M
121	OA	Fat pad	knee	Newcastle	69	F
122	OA	Cartilage	hip	Newcastle	55	M
123	OA	Fat pad	knee	Newcastle	72	M
124	OA	Cartilage & Fat pad	knee	Newcastle	53	F
125	OA	Cartilage	knee	Newcastle	70	M
126	OA	Cartilage & Fat pad	knee	Newcastle	75	M
127	OA	Synovium	hip	Newcastle	91	F
128	OA	Cartilage	hip	Newcastle	61	M
129	OA	Cartilage	knee	Newcastle	75	M
130	OA	Cartilage & Fat pad	knee	Newcastle	76	M
131	OA	Cartilage	hip	Newcastle	66	F

132	OA	Cartilage	hip	Newcastle	77	M
133	OA	Cartilage	hip	Newcastle	65	F
134	OA	Bone	hip	Newcastle	65	F
135	OA	Bone	knee	Newcastle	72	M
136	OA	Bone	hip	Newcastle	71	M
137	OA	Cartilage	knee	Newcastle	79	F
138	OA	Cartilage	hip	Newcastle	64	F
139	OA	Bone	knee	Newcastle	82	M
140	OA	Bone	hip	Newcastle	66	M
141	OA	Bone	knee	Newcastle	68	M
142	OA	Bone	knee	Newcastle	75	M
143	OA	Bone	knee	Newcastle	57	F
144	OA	Cartilage	knee	Newcastle	58	F
145	OA	Fat pad	knee	Newcastle	60	M
146	OA	Cartilage	hip	Newcastle	79	M
147	OA	Fat pad	knee	Newcastle	79	M
148	OA	Fat pad	knee	Newcastle	82	M
149	OA	Cartilage	hip	Newcastle	62	F
150	OA	Bone	hip	Newcastle	78	F
151	OA	Cartilage	hip	Newcastle	88	M
152	OA	Cartilage	knee	Newcastle	82	F
153	OA	Cartilage	knee	Newcastle	63	F
154	OA	Bone	knee	Newcastle	58	M
155	OA	Cartilage	knee	Newcastle	59	F
156	OA	Cartilage	knee	Newcastle	66	M
157	OA	Cartilage	knee	Newcastle	76	M
158	OA	Cartilage & Fat pad & Bone	knee	Newcastle	81	F
159	OA	Cartilage	hip	Newcastle	71	M
160	OA	Cartilage	hip	Newcastle	47	M
161	OA	Cartilage & Fat pad & Synovium	knee	Newcastle	76	M
162	OA	Fat pad & Synovium	knee	Newcastle	68	F
163	OA	Cartilage & Fat pad & Synovium & Bone	knee	Newcastle	63	F
164	OA	Bone	hip	Newcastle	72	M
165	OA	Cartilage & Fat pad & Synovium	knee	Newcastle	59	F
166	OA	Cartilage	hip	Newcastle	81	F
167	OA	Cartilage & Fat pad & Synovium	knee	Newcastle	54	F
168	OA	Bone	knee	Newcastle	78	F
169	OA	Cartilage	hip	Newcastle	73	F
170	OA	Cartilage & Fat pad & Synovium	knee	Newcastle	70	F
171	OA	Cartilage	hip	Newcastle	71	M
172	OA	Cartilage	hip	Newcastle	69	F
173	OA	Cartilage & Fat pad & Synovium & Bone	knee	Newcastle	81	M
174	OA	Cartilage	knee	Newcastle	60	F
175	OA	Cartilage	hip	Newcastle	66	F
176	OA	Cartilage	hip	Newcastle	81	M
177	OA	Bone	hip	Newcastle	61	F
178	OA	Bone	hip	Newcastle	64	F
179	OA	Cartilage	knee	Newcastle	71	M
180	OA	Cartilage	hip	Newcastle	67	F
181	OA	Bone	knee	Newcastle	57	F
182	OA	Bone	knee	Newcastle	64	F
183	OA	Bone	knee	Newcastle	54	M
184	OA	Bone	hip	Newcastle	62	M
185	OA	Bone	knee	Newcastle	73	M
186	OA	Bone	knee	Newcastle	86	M
187	OA	Bone	knee	Newcastle	54	F
188	OA	Bone	knee	Newcastle	75	F
189	OA	Bone	knee	Newcastle	78	M
190	OA	Bone	knee	Newcastle	51	M
191	OA	Bone	hand	Newcastle	59	F
192	OA	Fat pad	knee	Newcastle	75	M
193	OA	Fat pad	knee	Newcastle	73	F
194	OA	Fat pad	knee	Newcastle	82	F
195	OA	Fat pad	knee	Newcastle	62	F
196	OA	Fat pad	knee	Newcastle	82	F
197	OA	Fat pad	knee	Newcastle	65	F
209	OA	Osteochondral	hand/trapezium	Newcastle	67	F
210	OA	Osteochondral	hand/trapezium	Newcastle	71	F
211	OA	Osteochondral	hand/trapezium	Newcastle	70	F

212	OA	Osteochondral	hand/trapezium	Newcastle	82	F
213	OA	Osteochondral	hand/trapezium	Newcastle	58	F
214	OA	Osteochondral	hand/trapezium	Newcastle	46	F
215	OA	Osteochondral	hand/trapezium	Newcastle	52	F
216	OA	Osteochondral	hand/trapezium	Newcastle	56	F
217	OA	Osteochondral	hand/trapezium	Newcastle	66	F
218	OA	Osteochondral	hand/trapezium	Newcastle	60	M
219	OA	Osteochondral	hand/trapezium	Newcastle	61	M
220	OA	Osteochondral	hand/trapezium	Newcastle	66	M
222	OA	Osteochondral	hand/trapezium	Oxford	67	F
223	OA	Osteochondral	hand/trapezium	Oxford	50	F
224	OA	Osteochondral	hand/trapezium	Oxford	75	F
225	OA	Osteochondral	hand/trapezium	Oxford	65	M
226	OA	Osteochondral	hand/trapezium	Oxford	54	F

Details of the patients from whom chondrocytes were isolated for use in AEI, transcript stability and RNAi experiments

Patient number	Phenotype	Tissue	Joint site affected	Collection site	Age at surgery (years)	Sex
198	OA	Cartilage	knee	Newcastle	57	F
199	OA	Cartilage	knee	Newcastle	58	F
200	OA	Cartilage	knee	Newcastle	61	F
201	OA	Cartilage	knee	Newcastle	77	F
202	OA	Cartilage	knee	Newcastle	67	F
203	OA	Cartilage	knee	Newcastle	58	M
204	OA	Cartilage	knee	Newcastle	60	M
205	OA	Cartilage	knee	Newcastle	72	F
206	OA	Cartilage	hip	Newcastle	85	F
207	OA	Cartilage	hip	Newcastle	54	F
208	OA	Cartilage	knee	Newcastle	58	F
221	OA	Cartilage	knee	Newcastle	70	F

Details of the patients used for generating RNA-seq data

Patient ID	Phenotype	Tissue	Joint site affected	Collection site	Age at surgery (years)	Sex
227	OA	Cartilage	hip	Newcastle	66	F
228	OA	Cartilage	hip	Newcastle	76	F
229	OA	Cartilage	hip	Newcastle	83	F
230	OA	Cartilage	hip	Newcastle	78	F
231	OA	Cartilage	hip	Newcastle	83	F
232	OA	Cartilage	hip	Newcastle	60	F
233	OA	Cartilage	hip	Newcastle	55	F
234	OA	Cartilage	hip	Newcastle	82	F
235	OA	Cartilage	hip	Newcastle	72	F
236	OA	Cartilage	hip	Newcastle	72	F
237	NOF	Cartilage	hip	Newcastle	85	F
238	NOF	Cartilage	hip	Newcastle	71	F
239	NOF	Cartilage	hip	Newcastle	81	F
240	NOF	Cartilage	hip	Newcastle	72	F
241	NOF	Cartilage	hip	Newcastle	84	F
242	NOF	Cartilage	hip	Newcastle	94	F
