# Supplementary material for: Functional Characterization of the Osteoarthritis Genetic Risk Residing at ALDH1A2 Identifies rs12915901 as a Key Target Variant
Source: Arthritis Rheumatol. 2018 Aug 23;70(10):1577–87. doi: 10.1002/art.40545 (PMC6175168; doi:10.1002/art.40545)
Supplement: Supplementary file 5 — Supplementary Table 2 [file ART-70-1577-s005.docx]

| Gene | Forward primer (5'-3') | Reverse primer (5'-3') | Roche Probe Library probe number |
| --- | --- | --- | --- |
| *ALDH1A2* | CCACAGTGTTTTCCAACGTC | TCCTGAACAGGGCCAAAG | #63 |
| *RARA* | GCCTGCCTGGACATCCTG | AGCCAGCGTTGTGCATCT | #15 |
| *RARB* | TCGGCACACTGCTCAATC | GAAGCAGGGTTTGTACACTCG | #69 |
| *RARG* | GCTTTACAGGGCTCAGCATT | CTTGTGCAGATACGCAGCAT | #27 |
| *RXRA* | ACATGCAGATGGACAAGACG | TCGAGAGCCCCTTGGAGT | #26 |
| *RXRB* | AGCTCCCCCAGGATTCTC | CAGGGAGTGACACTGTTGAGTTA | #66 |
| *CRABP2* | CGAGGAATTGCTCAAAGTGC | TCCTGTTTGATCTCCACTGCT | #73 |
| *CYP26B1* | CCTGCCCTTCAGTGGCTA | GTAGTCCTTGCCCTGTGTGC | #7 |

**Supplemental Table 2.** Primers used for qRT-PCR using the Roche probe library and custom designed assays. Assays were designed using the Roche Probe Library Design Centre. The relevant probe number is listed
