# Supplementary material for: Functional Characterization of the Osteoarthritis Genetic Risk Residing at ALDH1A2 Identifies rs12915901 as a Key Target Variant
Source: Arthritis Rheumatol. 2018 Aug 23;70(10):1577–87. doi: 10.1002/art.40545 (PMC6175168; doi:10.1002/art.40545)
Supplement: Supplementary file 6 — Supplementary Table 3 [file ART-70-1577-s006.docx]

**Supplemental Table 3.** Primers used for allelic expression imbalance (AEI) analysis and genotyping. rs3204689 was genotyped by a restriction fragment length polymorphism (RFLP) assay using *SfcI* whereas rs4238326 was genotyped by pyrosequencing. Btn, biotin tag at the 5' end of the primer. n/a, not applicable

| Target SNP | Assay | Forward primer (5'-3') | Reverse primer (5'-3') | Sequencing primer (5'-3') |
| --- | --- | --- | --- | --- |
| rs3204689 | AEI | Btn-AATGAAAATGCCAGGAGCTGGTAC | TGCACATGATGACTTCCAGTCTCT | TCCCAGGTTCTTACTACA |
| rs3204689 | Genotyping | TTCAAAGCAAAACATGAAATGG | AGCTCAGAAGCGGTGAACTG | n/a |
| rs4238326 | Genotyping | ACGGTTTTTGTTTCCTGTTGTGA | Btn-TTCCTAACCAAAGGAGCCTCTACT | CATACTTTAACAGCCAAGTC |
