# Supplementary material for: Functional Characterization of the Osteoarthritis Genetic Risk Residing at ALDH1A2 Identifies rs12915901 as a Key Target Variant
Source: Arthritis Rheumatol. 2018 Aug 23;70(10):1577–87. doi: 10.1002/art.40545 (PMC6175168; doi:10.1002/art.40545)
Supplement: Supplementary file 8 — Supplementary Table 5 [file ART-70-1577-s008.docx]

| SNP | Allele | Forward primer (5'-3') | Reverse primer (5'-3') |
| --- | --- | --- | --- |
| rs4646636 | T  C | TGTCTAGATTGCATGTTTGTTTGTTTGTTTG  TGTCTAGATTGCATGCTTGTTTGTTTGTTTG | CAAACAAACAAACAAACATGCAATCTAGACA  CAAACAAACAAACAAGCATGCAATCTAGACA |
| rs12915901 | C  T | TTTTGCTTTCTACTTCCGCCTTCTGTTTACA  TTTTGCTTTCTACTTTCGCCTTCTGTTTACA | TGTAAACAGAAGGCGGAAGTAGAAAGCAAAA  TGTAAACAGAAGGCGAAAGTAGAAAGCAAAA |
| rs4646563 | A  G | CCACTACATGGGCCTATGTTACCATGTGGAA  CCACTACATGGGCCTGTGTTACCATGTGGAA | TTCCACATGGTAACATAGGCCCATGTAGTGG  TTCCACATGGTAACACAGGCCCATGTAGTGG |
| rs4646586 | G  T | ACTTCTGCTCCGTTTGCCCTGTCATTCTGTG  ACTTCTGCTCCGTTTTCCCTGTCATTCTGTG | CACAGAATGACAGGGCAAACGGAGCAGAAGT  CACAGAATGACAGGGAAAACGGAGCAGAAGT |
| rs11071365 | C  T | TTACATAACTTACTACGATATGGTGTTGGAG  TTACATAACTTACTATGATATGGTGTTGGAG | CTCCAACACCATATCGTAGTAAGTTATGTAA  CTCCAACACCATATCATAGTAAGTTATGTAA |
| rs11071366 | T  A | TCTCGCCAAAAATCATATGTTCAAATCCTAA  TCTCGCCAAAAATCAAATGTTCAAATCCTAA | TTAGGATTTGAACATATGATTTTTGGCGAGA  TTAGGATTTGAACATTTGATTTTTGGCGAGA |
| rs4646571 | A  G | TCAAAGCCTTCTTCAACCTTAGGATATGCAT  TCAAAGCCTTCTTCAGCCTTAGGATATGCAT | ATGCATATCCTAAGGTTGAAGAAGGCTTTGA  ATGCATATCCTAAGGCTGAAGAAGGCTTTGA |
| rs4646572 | A  G | AGACATTAACTTCTAATTCAACTGAAGATGC  AGACATTAACTTCTAGTTCAACTGAAGATGC | GCATCTTCAGTTGAATTAGAAGTTAATGTCT  GCATCTTCAGTTGAACTAGAAGTTAATGTCT |

**Supplemental Table 5.** Primers used to create the EMSA probes targeting both alleles of each SNP analysed. The polymorphic base is underlined in each primer
