# Supplementary material for: Functional Characterization of the Osteoarthritis Genetic Risk Residing at ALDH1A2 Identifies rs12915901 as a Key Target Variant
Source: Arthritis Rheumatol. 2018 Aug 23;70(10):1577–87. doi: 10.1002/art.40545 (PMC6175168; doi:10.1002/art.40545)
Supplement: Supplementary file 9 — Supplementary Table 6 [file ART-70-1577-s009.docx]

| Transcription factor targeted | Forward primer (5'-3') | Reverse primer (5'-3') |
| --- | --- | --- |
| Random competitor | TAGCACCTGACTGACGTCTGAGTACGTAG | CTACGTACTCAGACGTCAGTCAGGTGCTA |
| ETS | TAGCACCTGACC**GGAA**GTTGAGTACGTAG | CTACGTACTCAAC**TTCC**GGTCAGGTGCTA |

**Supplemental Table 6.** Primers used to create a random competitor EMSA probe and a probe targeting ETS transcription factors. The consensus transcription factor binding site for ETS (5'-GGAA-3') is highlighted in bold
