# Supplementary material for: Functional Characterization of the Osteoarthritis Genetic Risk Residing at ALDH1A2 Identifies rs12915901 as a Key Target Variant
Source: Arthritis Rheumatol. 2018 Aug 23;70(10):1577–87. doi: 10.1002/art.40545 (PMC6175168; doi:10.1002/art.40545)
Supplement: Supplementary file 10 — Supplementary Table 7 [file ART-70-1577-s010.docx]

| Name | ID | Protein coding |
| --- | --- | --- |
| ALDH1A2-201 | ENST00000249750.8 | Yes |
| ALDH1A2-202 | ENST00000347587.7 | Yes |
| ALDH1A2-203 | ENST00000430119.6 | No |
| ALDH1A2-204 | ENST00000537372.5 | Yes |
| ALDH1A2-212 | ENST00000558595.5 | No |
| ALDH1A2-215 | ENST00000559266.5 | No |
| ALDH1A2-217 | ENST00000559517.5 | Yes |
| ALDH1A2-221 | ENST00000560312.5 | No |
| ALDH1A2-223 | ENST00000560923.5 | No |

**Supplemental Table 7.** The Ensemble names and identification (ID) numbers for the nine *ALDH1A2* transcript isoforms expressed in the cartilage RNA-seq data
