# Supplementary material for: Functional Characterization of the Osteoarthritis Genetic Risk Residing at ALDH1A2 Identifies rs12915901 as a Key Target Variant
Source: Arthritis Rheumatol. 2018 Aug 23;70(10):1577–87. doi: 10.1002/art.40545 (PMC6175168; doi:10.1002/art.40545)
Supplement: Supplementary file 11 — Supplementary Table 8 [file ART-70-1577-s011.docx]

**Supplemental Table 8.** The additional RA pathway genes examined in the RNA-seq dataset

| Gene | Full protein name | Function in RA pathway |
| --- | --- | --- |
| *RARA* | Retinoic acid receptor alpha | Signalling |
| *RARB* | Retinoic acid receptor beta | Signalling |
| *RARG* | Retinoic acid receptor gamma | Signalling |
| *RXRA* | Retinoic acid receptor RXR-alpha | Signalling |
| *RXRB* | Retinoic acid receptor RXR-beta | Signalling |
| *CRABP2* | Cellular retinoic acid-binding protein 2 | Transport |
| *STRA6* | Stimulated by retinoic acid gene 6 protein homolog | Transport |
| *CYP26B1* | Cytochrome P450 26B1 | Metabolism |
| *ALDH1A3* | Aldehyde dehydrogenase family 1 member A3 | Metabolism |
| *RARRES1* | Retinoic acid receptor responder protein 1 | Response |
| *RAI1* | Retinoic acid-induced protein 1 | Response |
| *RAI2* | Retinoic acid-induced protein 2 | Response |
