# Supplementary material for: Functional Characterization of the Osteoarthritis Genetic Risk Residing at ALDH1A2 Identifies rs12915901 as a Key Target Variant
Source: Arthritis Rheumatol. 2018 Aug 23;70(10):1577–87. doi: 10.1002/art.40545 (PMC6175168; doi:10.1002/art.40545)
Supplement: Supplementary file 12 — Supplementary Table 9 [file ART-70-1577-s012.docx]

| SNP | Coordinate (hg38) | r² relative to rs3204689 | D' relative to rs3204689 | Reference  allele | Risk  allele | Location | RegulomeDB score | Taken forward for luciferase analysis? |
| --- | --- | --- | --- | --- | --- | --- | --- | --- |
| rs4775005 | 15:57915967 | 0.8 | 0.92 | G | A | Intergenic | 3a | Yes |
| rs8033270 | 15:57925591 | 0.86 | 0.93 | G | C | Intergenic | no supporting data | No |
| rs11852835 | 15:57936401 | 0.88 | 0.97 | A | G | Intergenic | 5 | Yes |
| rs1579744 | 15:57937325 | 0.88 | 0.97 | G | A | Intergenic | 6 | Yes |
| rs17820823 | 15:57947160 | 0.96 | 0.99 | A | G | Intergenic | no supporting data | No |
| rs7178497 | 15:57948135 | 0.95 | 0.99 | C | T | Intergenic | 5 | Yes |
| rs11071356 | 15:57948320 | 0.95 | 0.99 | T | G | Intergenic | 5 | Yes |
| rs10851630 | 15:57949950 | 0.95 | 0.99 | C | T | Intergenic | 5 | Yes |
| rs10851631 | 15:57949995 | 0.95 | 0.99 | C | T | Intergenic | 5 | Yes |
| rs12907038 | 15:57951834 | 0.97 | 0.99 | C | G | Intergenic | 5 | Yes |
| rs7495968 | 15:57951905 | 0.99 | 1 | C | G | Intergenic | 5 | Yes |
| rs9325 | 15:57953788 | 0.96 | 1 | T | A | *ALDH1A2* 3' UTR | no supporting data | No |
| **rs3204689** | **15:57954604** | **n/a** | **n/a** | **G** | **C** | ***ALDH1A2* 3' UTR** | **no supporting data** | **No** |
| rs4646640 | 15:57955365 | 0.99 | 1 | C | G | *ALDH1A2* intronic | 6 | Yes |
| rs12910113 | 15:57956175 | 0.99 | 1 | A | C | *ALDH1A2* intronic | no supporting data | No |
| rs4646638 | 15:57956585 | 0.98 | 0.99 | A | G | *ALDH1A2* intronic | no supporting data | No |
| rs11855259 | 15:57957179 | 0.95 | 0.98 | C | G | *ALDH1A2* intronic | 5 | Yes |
| rs4646636 | 15:57958836 | 0.94 | 0.98 | A | G | *ALDH1A2* intronic | 6 | Yes |
| rs7165247 | 15:57959588 | 0.93 | 0.98 | T | C | *ALDH1A2* intronic | 5 | Yes |
| rs7170896 | 15:57960195 | 0.92 | 0.98 | T | A | *ALDH1A2* intronic | 5 | Yes |
| rs3784263 | 15:57960864 | 0.9 | 0.96 | C | T | *ALDH1A2* intronic | no supporting data | No |
| rs3784262 | 15:57960908 | 0.94 | 0.98 | T | C | *ALDH1A2* intronic | no supporting data | No |
| rs3784260 | 15:57961071 | 0.91 | 0.96 | T | G | *ALDH1A2* intronic | 6 | Yes |
| rs4646629 | 15:57963017 | 0.94 | 0.99 | A | G | *ALDH1A2* intronic | 3a | Yes |
| rs4646628 | 15:57963084 | 0.92 | 0.96 | T | C | *ALDH1A2* intronic | 2b | Yes |
| rs4646627 | 15:57963107 | 0.94 | 0.99 | A | G | ALDH1A2 intronic | 3a | Yes |
| rs4646622 | 15:57965001 | 0.94 | 0.99 | A | G | *ALDH1A2* intronic | no supporting data | No |
| rs4646620 | 15:57966043 | 0.9 | 0.96 | G | A | *ALDH1A2* intronic | no supporting data | No |
| rs4646619 | 15:57966144 | 0.94 | 0.99 | A | G | *ALDH1A2* intronic | 6 | Yes |
| rs7164408 | 15:57967138 | 0.92 | 0.99 | A | G | *ALDH1A2* intronic | no supporting data | No |
| rs4646617 | 15:57969001 | 0.89 | 0.95 | ACAC | A | *ALDH1A2* intronic | no supporting data | No |
| rs12903792 | 15:57969082 | 0.89 | 0.95 | C | T | *ALDH1A2* intronic | no supporting data | No |
| rs4646616 | 15:57969151 | 0.89 | 0.95 | G | A | *ALDH1A2* intronic | no supporting data | No |
| rs4646612 | 15:57972879 | 0.89 | 0.99 | C | T | *ALDH1A2* intronic | no supporting data | No |
| rs4646611 | 15:57972885 | 0.94 | 0.99 | T | C | *ALDH1A2* intronic | no supporting data | No |
| rs35511675 | 15:57974780 | 0.94 | 0.99 | T | A | *ALDH1A2* intronic | 6 | Yes |
| rs66725070 | 15:57975218 | 0.92 | 0.98 | GACAT | G | *ALDH1A2* intronic | 4 | Yes |
| rs12903551 | 15:57976174 | 0.94 | 0.98 | C | T | *ALDH1A2* intronic | no supporting data | No |
| rs11630835 | 15:57977264 | 0.93 | 0.98 | C | T | *ALDH1A2* intronic | 5 | Yes |
| rs12903474 | 15:57984549 | 0.94 | 0.97 | C | A | *ALDH1A2* intronic | no supporting data | No |
| rs12908953 | 15:57985614 | 0.92 | 0.97 | A | G | *ALDH1A2* intronic | no supporting data | No |
| rs12915901 | 15:57987234 | 0.92 | 0.98 | G | A | *ALDH1A2* intronic | 6 | Yes |
| rs34871384 | 15:57987676 | 0.92 | 0.98 | C | A | *ALDH1A2* intronic | no supporting data | No |
| rs12148907 | 15:58004252 | 0.91 | 0.94 | T | A | *ALDH1A2* intronic | no supporting data | No |
| rs1372368 | 15:58005165 | 0.88 | 0.94 | C | T | *ALDH1A2* intronic | 5 | Yes |
| rs4646593 | 15:58010269 | 0.88 | 0.95 | A | G | *ALDH1A2* intronic | 5 | Yes |
| rs4646586 | 15:58013146 | 0.88 | 0.95 | C | A | *ALDH1A2* intronic | 5 | Yes |
| rs12910752 | 15:58022481 | 0.88 | 0.95 | G | A | *ALDH1A2* intronic | 6 | Yes |
| rs12911071 | 15:58022650 | 0.88 | 0.95 | G | A | *ALDH1A2* intronic | no supporting data | No |
| rs113890824 | 15:58024169 | 0.84 | 0.93 | GGAAGA | G | *ALDH1A2* intronic | 6 | Yes |
| rs12901462 | 15:58024289 | 0.89 | 0.95 | A | G | *ALDH1A2* intronic | 6 | Yes |
| rs4369598 | 15:58026405 | 0.89 | 0.95 | G | T | *ALDH1A2* intronic | 3a | Yes |
| rs11852540 | 15:58029266 | 0.88 | 0.94 | A | G | *ALDH1A2* intronic | no supporting data | No |
| rs12148093 | 15:58036742 | 0.86 | 0.93 | A | T | *ALDH1A2* intronic | 5 | Yes |
| rs4646563 | 15:58061137 | 0.84 | 0.92 | T | C | *ALDH1A2* intronic | no supporting data | Yes |

**Supplemental Table 9.** The 54 SNPs in LD (r^2^ > 0.8) with rs3204689. Pairwise LD values (r^2^ and Dʹ) relative to rs3204689 are listed, as is the physical coordinate location of each SNP using hg38. rs3204689 is bold highlighted. A key to the RegulomeDB scores can be found in Fig. S1
