# Supplementary material for: Functional Characterization of the Osteoarthritis Genetic Risk Residing at ALDH1A2 Identifies rs12915901 as a Key Target Variant
Source: Arthritis Rheumatol. 2018 Aug 23;70(10):1577–87. doi: 10.1002/art.40545 (PMC6175168; doi:10.1002/art.40545)
Supplement: Supplementary file 13 — Supplementary Table 10 [file ART-70-1577-s013.docx]

| SNP | Coordinate (hg38) | r² relative to rs4238326 | D' relative to rs4238326 | Reference  allele | Risk  allele | Location | RegulomeDB score | Taken forward for luciferase analysis? |
| --- | --- | --- | --- | --- | --- | --- | --- | --- |
| rs10851633 | 15:58030867 | 0.98 | 0.99 | C | T | *ALDH1A2* intronic | no supporting data | No |
| rs11071365 | 15:58041928 | 1 | 1 | G | A | *ALDH1A2* intronic | 3a | Yes |
| rs11071366 | 15:58042046 | 0.99 | 1 | A | T | *ALDH1A2* intronic | 3a | Yes |
| **rs4238326** | **15:58043802** | **n/a** | **n/a** | **T** | **C** | ***ALDH1A2* intronic** | **4** | **Yes** |
| rs35246600 | 15:58044228 | 1 | 1 | T | A | *ALDH1A2* intronic | no supporting data | No |
| rs4646576 | 15:58046800 | 0.99 | 1 | A | T | *ALDH1A2* intronic | 5 | Yes |
| rs4646572 | 15:58050823 | 0.84 | 1 | T | C | *ALDH1A2* intronic | 5 | Yes |
| rs4646571 | 15:58050939 | 0.81 | 0.98 | T | C | *ALDH1A2* intronic | 5 | Yes |
| rs4646568 | 15:58052092 | 0.98 | 1 | C | T | *ALDH1A2* intronic | 6 | Yes |

**Supplemental Table 10.** The eight SNPs in LD (r^2^ > 0.8) with rs4238326. Pairwise LD values (r^2^ and Dʹ) relative to rs4238326 are listed, as is the physical coordinate location of each SNP using hg38. rs4238326 is bold highlighted. A key to the RegulomeDB scores can be found in Fig. S1
